# Microarray analysis identifies a common set of cellular genes modulated by different HCV replicon clones

**DOI:** 10.1186/1471-2164-9-309

**Published:** 2008-06-30

**Authors:** Anna Rita Ciccaglione, Cinzia Marcantonio, Elena Tritarelli, Paola Tataseo, Alessandro Ferraris, Roberto Bruni, Bruno Dallapiccola, Germano Gerosolimo, Angela Costantino, Maria Rapicetta

**Affiliations:** 1 Department of Infectious, Parasitic and Immunomediated Diseases, Istituto Superiore di Sanità, Rome, Italy; 2 ASL Avezzano-Sulmona, Transfusional Medicine and Molecular Biology Laboratory, Sulmona, Italy; 3 IRCCS CSS-Mendel Institute and Dept. of Experimental Medicine and Pathology, Sapienza University, Rome, Italy

## Abstract

**Background:**

Hepatitis C virus (HCV) RNA synthesis and protein expression affect cell homeostasis by modulation of gene expression. The impact of HCV replication on global cell transcription has not been fully evaluated. Thus, we analysed the expression profiles of different clones of human hepatoma-derived Huh-7 cells carrying a self-replicating HCV RNA which express all viral proteins (HCV replicon system).

**Results:**

First, we compared the expression profile of HCV replicon clone 21-5 with both the Huh-7 parental cells and the 21-5 cured (21-5c) cells. In these latter, the HCV RNA has been eliminated by IFN-α treatment. To confirm data, we also analyzed microarray results from both the 21-5 and two other HCV replicon clones, 22-6 and 21-7, compared to the Huh-7 cells. The study was carried out by using the Applied Biosystems (AB) Human Genome Survey Microarray v1.0 which provides 31,700 probes that correspond to 27,868 human genes. Microarray analysis revealed a specific transcriptional program induced by HCV in replicon cells respect to both IFN-α-cured and Huh-7 cells. From the original datasets of differentially expressed genes, we selected by Venn diagrams a final list of 38 genes modulated by HCV in all clones. Most of the 38 genes have never been described before and showed high fold-change associated with significant p-value, strongly supporting data reliability. Classification of the 38 genes by Panther System identified functional categories that were significantly enriched in this gene set, such as histones and ribosomal proteins as well as extracellular matrix and intracellular protein traffic. The dataset also included new genes involved in lipid metabolism, extracellular matrix and cytoskeletal network, which may be critical for HCV replication and pathogenesis.

**Conclusion:**

Our data provide a comprehensive analysis of alterations in gene expression induced by HCV replication and reveal modulation of new genes potentially useful for selection of antiviral targets.

## Background

Infection with hepatitis C virus (HCV) represents the major cause of liver disease, affecting more than 170 million individuals worldwide. After a sub-clinical phase, greater than 80% of patients progress to persistent HCV infection which is the leading cause of chronic liver disease associated with cirrhosis and hepatocellular carcinoma [1,2].

Research on HCV replication and pathogenesis has been hampered by the lack of reproducible *in vitro *methods of HCV infection. To overcome these restrictions, selectable HCV replicons in human hepatoma-derived Huh7 cells, which contain self-replicating HCV RNA and express all viral proteins, were developed [3-6]. These systems have been largely used for the study of HCV translation and RNA replication revealing important processes of virus-host interactions [7-12]. HCV proteins have been proposed to be involved in a wide range of activities, including cell signaling, transcriptional modulation, transformation, apoptosis, oxidative stress, membrane rearrangement, vesicular trafficking and immune response [13-21]. Recently, cell culture models that release HCV viral particles have been developed providing new possibilities for molecular studies of the HCV life cycle and virus-cell interactions [22-24]. Interestingly, a set of 26 human genes that modulate virus production have been identified by using a siRNA screening approach, suggesting the possibility to target host genes for antiviral therapy [25].

Microarray technology provides the opportunity to study HCV-host interactions at genomic level and identify novel genes relevant for HCV infection. Despite a large body of evidences suggesting that HCV affects cell homeostasis, a first microarray analysis revealed that HCV replicon exerts a minimal effect on host cell expression profile. On the other hand, this study indicated that a common transcriptional response to HCV could be detected in different clones of replicon cells. This response, however, involves only a limited number of mRNAs that show minor changes in the expression level and remains to be more fully elucidated [26].

Microarray is a rapidly implementing technology and the commercially available platforms show unique genomic targets and use different methodologies for probe design and for detection of signals, resulting in different level of sensitivity [27,28]. Moreover, the final list of human genes has yet to be determined and reference sequences are periodically modified. On this basis, to identify cellular genes that are modulated by HCV RNA replication, we analyzed the expression profile of cell lines carrying a full-length HCV genome by a new microarray platform developed by the Applied Biosystems. To enhance the sensitivity to low abundance transcripts, this platform employs 60-mers oligonucleotides and a chemiluminescence-based approach to detection, with reduced background noise relative to standard fluorescent systems.

Present data indicated that the AB microarray shows the sensitivity to detect a higher number of cellular genes modulated by HCV in replicon cells respect to both cells cultured with IFN α, a treatment which has been shown to eliminate self-replicating HCV RNA [9], and Huh-7 cells. From the original datasets of differentially expressed genes, we selected 38 genes that show a concordant expression in three different HCV replicon clones and are potentially implicated in HCV infection. Our study also confirmed the involvement of previously identified cellular processes in HCV replication, and provided archived microarray databases useful for selection of new targets of antiviral therapy.

## Results

### Modulation of gene expression in 21-5 cells carrying a full-length HCV replicon

To determine the impact of HCV protein synthesis and RNA replication on host cell transcription, we compared the expression profile of the 21-5 cell line, harbouring a full-length HCV genome, with the 21-5 cured cell line (21-5c) and the Huh-7 parental cell line. The study was carried out by using the Applied Biosystems (AB) Human Genome Survey Microarray v1.0 which provides 31,700 probes that correspond to 27,868 human genes. The experimental design included two biological replicates (cells seeded in different plates) for each cell line. In addition, as hybridization of the same RNA extract with two different arrays (technical replicates) may significantly increase the number of identified genes by AB platform [27], we also included two technical replicates for each biological replicate for a total of twelve microarray experiments. The number of differentially expressed genes in the 21-5 *vs*. 21-5c (dataset 1) and 21-5 *vs*. Huh-7 (dataset 2) comparisons was evaluated by filtering data using a signal/noise (S/N) ratio > 3.0 in 75% of replicates in at least one tested sample group (either 21-5 or 21-5c and either 21-5 or Huh-7) and p ≤ 0.05. The 21-5 *vs*. 21-5c and 21-5 *vs*. Huh-7 comparisons showed 733 and 865 differentially expressed probes, respectively (dataset 1 and dataset 2 in Table 1). After subtraction for probes considered to be obsolete and genes now recognised to be pseudogenes, the number was reduced to 690 and 810 in dataset 1 and 2, respectively (Table 1, see Current probes). Probes showing a fold-change (FC) ≥ 2 were 288 for comparison 1 (112, up-regulated; 176, down-regulated). In comparison 2, we observed 352 probes with a fold-change ≥ 2 (250, up-regulated; 102, down-regulated). These findings indicated that microarray analysis with the AB platform showed enough sensitivity to reveal a specific transcriptional program induced by HCV in replicon cells.

**Table 1:** Number of probes with significantly different expression (p ≤ 0.05)

Probes	Comparisons	
	
	21-5 *vs*. 21-5c (dataset 1)	21-5 *vs*. Huh-7 (dataset 2)
Total	733	865
Current *	690	810
FC ≥ 2	288	352
Up	112	250
Down	176	102

* Probe set after the exclusion of obsolete genes and pseudogenes.

### Functional classification of genes altered by HCV in 21-5 replicon clone

**Figure 1:**
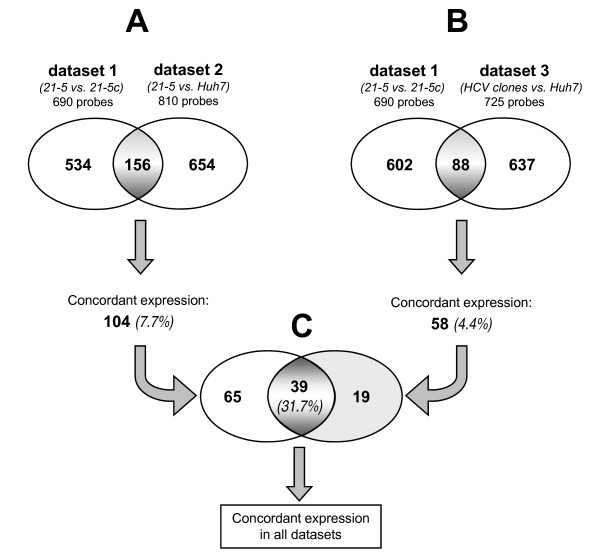
(A) Venn diagram of probes differentially expressed in 21-5 *vs*. 21-5c (dataset 1) and 21-5 *vs*. Huh-7 (dataset 2) comparisons. The number of probes differentially expressed in both comparisons was 156, and 104 (7.7%) out of 1344 total probes showed concordant expression (up-regulated or down-regulated in both datasets). (B) Venn diagram of the differentially expressed probes in 21-5 *vs*. 21-5c (dataset 1) and HCV clones *vs*. Huh-7 (dataset 3) comparisons. The number of probes differentially expressed in both comparisons was 88, and 58 (4.4%) out of 1327 total probes showed concordant expression. (C) Venn diagram of the 104 and 58 probes identified 39 (31,7%) common probes out of 123.

To increase the likelihood to identify HCV-modulated genes, we selected probes showing a common expression pattern in both 1 and 2 datasets. Fig. 1A shows a Venn diagram of the number of probes altered by expression of HCV as detected in each comparison alone or in both. Out of the 690 probes detected in dataset 1, 156 of them overlapped with the 810 probes identified in dataset 2. Among these 156 probes, only those showing the same direction of regulation (i.e. probes identifying up-regulated or down-regulated genes in both datasets) were further selected. This resulted in a set of 104 (7.7%) probes with concordant expression out of 1,344 total probes (Fig. 1A). A list of the 104 probes, which exactly identified 103 genes, with name, symbol, accession number, probe ID, fold-changes (FC) and functional description is provided as supplementary information [see Additional file 1].

To classify genes into biological categories, we analyzed the Gene Ontology annotations of the 103 common genes with the Panther Protein Classification System [29]. As shown in Table 2, Panther System found several functional categories that were significantly enriched in this gene set compared to the entire NCBI reference list of human genome. We considered, as potentially interesting, only categories showing a p-value ≤ 0.05, as determined by the binomial statistic [30]. The 103 genes of the dataset were significantly classified by the Panther system in 18 biological processes (i.e., processes in which genes participate) and 14 molecular functions (i.e., biological functions of gene products). Furthermore, some of the identified categories were still significantly represented in the set of 103 genes even after the application of the Bonferroni correction for multiple testing, suggesting that the confidence for their over-representation is very strong (Table 2, see p-value with Bonferroni correction). Several genes are included in functional categories previously reported to be relevant for HCV, such as immunity and defense, oncogenesis, intracellular signaling cascade (NF-kappaB cascade), lipid, fatty acid and steroid metabolism and extracellular matrix providing a validation of our analysis. In addition, Panther identified genes in the categories of amino acid metabolism, carbohydrate metabolism and protein glycosylation that probably reflected a well described condition of endoplasmic reticulum (ER) stress due to accumulation of HCV proteins in the ER compartment [31-33]. Interestingly, new categories were also found in this gene set such as histones, signalling molecules, select calcium binding proteins and cell motility as well as genes involved in Glutamine glutamate conversion and Blood coagulation pathways (Table 2, see Pathways). Most of the described categories were even found in the wider set of 288 probes with fold-changes ≥ 2 from dataset 1 (21-5 *vs*. 21-5c) [see Additional file 2], supporting the biological relevance of this set of genes in HCV replication.

**Table 2:** Panther classification of biological processes, molecular functions and pathways significantly enriched in the set of 103 genes

	Observed Genes^•^	Expected Genes^••^	p-value^•••^	p-value with Bonferroni correction
**BIOLOGICAL PROCESSES**				
**Immunity and defense**	**14**	**5.39**	**9.78E-04**	**3.03E-02**
Stress response	4	0.82	9.44E-03	
T-cell mediated immunity	3	0.79	4.58E-02	
Granulocyte-mediated immunity	2	0.26	2.86E-02	
**Blood circulation and gas exchange activity**				
Other blood circulation and gas exchange activity	2	0.07	2.56E-03	
**Nucleoside. nucleotide and nucleic acid metabolism**				
Chromatin packaging and remodeling	5	0.97	3.01E-03	
**Protein metabolism and modification**				
Proteolysis	10	3.93	6.08E-03	
Protein glycosylation	3	0.79	4.52E-02	
**Developmental processes**				
Skeletal development	3	0.50	1.43E-02	
**Amino acid metabolism**	**4**	**0.94**	**1.51E-02**	
**Cell structure and motility**				
Cell motility	5	1.44	1.51E-02	
**Oncogenesis**				
Other oncogenesis	2	0.28	3.29E-02	
**Intracellular signaling cascade**				
NF-kappaB cascade	2	0.29	3.46E-02	
**Carbohydrate metabolism**				
Pentose-phosphate shunt	1	0.04	4.01E-02	
**Lipid. fatty acid and steroid metabolism**				
Fatty acid metabolism	3	0.76	4.19E-02	
**Homeostasis**	**3**	**0.80**	**4.70E-02**	
Growth factor homeostasis	1	0.03	2.82E-02	
Other homeostasis activities	2	0.28	3.29E-02	
				
**MOLECULAR FUNCTIONS**				
**Nucleic acid binding**				
Histone	5	0.35	3.08E-05	4.96E-03
**Extracellular matrix**	**7**	**1.57**	**1.06E-03**	**3.08E-02**
Extracellular matrix glycoprotein	4	0.45	1.18E-03	
Other extracellular matrix	2	0.15	1.03E-02	
**Signaling molecule**	**10**	**3.25**	**1.60E-03**	**4.65E-02**
Chemokine	2	0.22	2.09E-02	
**Transferase**	**9**			
Transaldolase	1	0.00	4.08E-03	
**Protease**	**6**	**2.28**	**2.72E-02**	
Serine protease	4	0.77	7.79E-03	
**Select calcium binding protein**	**4**	**1.12**	**2.65E-02**	
Annexin	2	0.29	3.46E-02	
**Miscellaneous function**		.0		
Storage protein	1	0.04	3.61E-02	
Myelin protein	1	0.05	4.79E-02	
**PATHWAYS**				
**Glutamine glutamate conversion**	**1**	**0.02**	**2.78E-05**	**2.02E-02**
**Blood coagulation**	**2**	**0.22**	**2.46E-02**	**2.17E-02**

^• ^Number of genes. in the dataset of 103 genes, that map to the indicated Panther classification categories

^•• ^Expected number of genes in the dataset of 103, based on the NCBI reference list of human genome

^••• ^p-value as determined by the binomial statistic. A cutoff of 0.05 has been applied

### Selection of genes modulated by different HCV replicon clones

One of the major concern in HCV studies is that the observed findings might be due to clonal selection of replicon cells rather than HCV replication and expression. To exclude that differences in gene expression were exclusive of the 21-5 clone, we carried out a second analysis, including microarray results from both the 21-5 clone and two other HCV replicon clones, 22-6 and 21-7, as well as the Huh-7 cell line. Overall, we analysed data from eleven arrays: four replicates (two technical replicates for each one of two biological replicates) from both 21-5 and Huh-7 cell lines, two biological replicates from clone 21-7 and one from clone 22-6, for a total of seven HCV arrays and four Huh-7 arrays. The number of differentially expressed genes was evaluated by filtering data using a S/N ratio > 3.0 in 75% of replicates in at least one tested sample group and p ≤ 0.05. The analysis revealed that 725 current probes were modulated in HCV clones as compared to Huh-7 cells (dataset 3) (Fig. 1B). As shown in supplementary information [see Additional file 3], a hierarchical clustering analysis performed on this probe list correctly grouped biological and technical replicates among either the HCV clones or the Huh-7 cells. In addition, this analysis clearly depicted that, in spite of some differences between HCV clones, a common transcriptional response to HCV is well detectable. It is reasonable to suppose that HCV replication level may influence only in minimal part the variation in gene expression observed between clones. In fact, only a slight variation (1.6 fold) in HCV RNA amount was reported: the average replication levels of HCV RNA in the cell clones 21-5, 22-6 and 21-7 ranged between 1.5 to 2.5 × 10^7 ^molecules per μg of total RNA [6], levels that were confirmed in our laboratory.

To further select, among the 725 probes, only genes which were confirmed also in the comparison with the cured cells, we overlapped datasets 3 and 1. Of the 725 probes detected in dataset 3, 88 of them were in common with the 690 probes identified in dataset 1. Among these 88 probes, 58 (4.4%) out of 1,327 total probes showed concordant expression in all different HCV replicon clones respect to both cured and Huh-7 cell lines (Fig. 1B). As a final step, we found that 39 probes were common to the sets of 58 and 104 probes identified above: thus, 39 (31.7%) out of 123 probes were confirmed in all three comparisons (Fig. 1C). The 38 genes identified by those 39 probes are included, marked by gene symbol in bold, in the larger list of 57 genes identified by the above 58 probes, reported as supporting information [see Additional file 4]. Although the used strategy of analysis seriously reduced the number of differentially expressed probes, it led to 4 to 8 fold enrichment of probes showing concordant expression in different datasets (7.7% and 4.4% *vs *31.7%, Fig. 1A, B and 1C). Modulation of 19 out of 58 probes did not reach statistical significance in dataset 2 (Fig. 1C) (the corresponding genes are marked by gene symbol in normal type in the list of 57 genes [see Additional file 4]). However, these genes were included in the subsequent ontological analysis as their modulation is highly significant in dataset 1 and 3.

**Figure 2:**
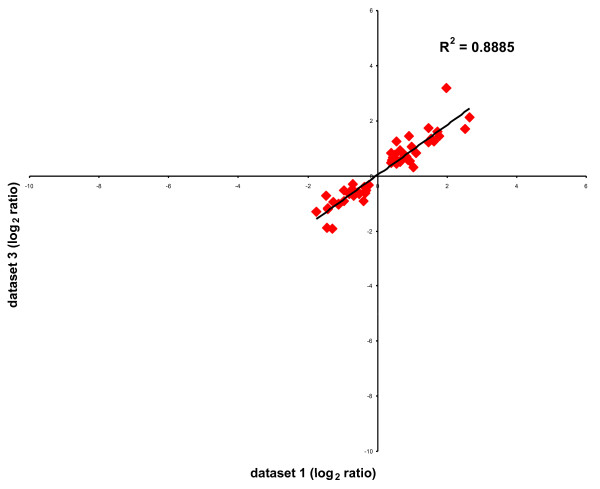
Scatterplot comparison of the log_2 _ratio of the 58 probes altered in the indicated datasets. Log_2 _ratios of genes from dataset 3 are plotted on the y-axis and from dataset 1 on the x-axis.

To directly compare the expression data from datasets 1 and 3, a scatter plot of the log_2 _fold-change was generated using the 58 probes with a significant fold-change in both comparisons (Fig. 2). As can be seen, the correlation is quite high (R^2 ^> 0.88), and consequently the confidence for the differential expression of these probes is strong.

### Biological functions of selected genes modulated by HCV in different replicon clones

A complete list of the 58 selected probes (32 up-regulated and 26 down-regulated), which exactly identified 57 genes, is provided in supplementary information [see Additional file 4]. Thirty-nine probes were confirmed in all datasets and identified 38 genes (marked by gene symbol in bold in Additional file 4). The list included name, symbol, accession number, probe ID, fold-changes (FC) and p-value in all datasets and functional description of the genes. The analysis of the FC in dataset 1 revealed that 19 (33%) genes showed a FC ≥ 2, while minor changes (FC 1.2–2) have been detected for the other genes. Compared with the NCBI reference list of human genome, this dataset showed a larger proportion of genes encoding nucleic acid binding proteins, such as histones and ribosomal proteins and genes involved in chromosome segregation and mRNA transcription (Table 3), possibly suggesting the induction by HCV of specific new pathways. In addition, genes associated with extracellular matrix constitution and intracellular protein traffic were represented much more abundantly in the dataset. Although two relevant categories, such as oxidative stress and lipid metabolism, were not significantly overrepresented, genes involved in these processes like DDIT3 and ELOVL6 were still present in the dataset [see Additional file 4].

**Table 3:** Panther classification of biological processes and molecular functions significantly enriched in the set of 57 genes

	Observed Genes^•^	Expected Genes^••^	p-value^•••^	p-value with Bonferroni correction
**BIOLOGICAL PROCESSES**				
**Nucleoside, nucleotide and nucleic acid ** **metabolism**	**14**	**7.62**	** 1.63E-02**	
Chromatin packaging and remodeling	6	0.54	1.75E-05	2.54E-03
Chromosome segregation	2	0.28	3.14E-02	
Other mRNA transcription	1	0.05	4.68E-02	
**Intracellular protein traffic**	**6**	**2.3**	**2.70E-02**	
**Molecular Functions**				
**Nucleic acid binding**	**16**	**6.5**	**4.82E-04**	**1.40E-02**
Histone	6	0.2	5.21E-08	8.38E-06
Ribosomal protein	5	1.06	4.19E-03	
**Etracellular matrix**	**5**	**0.88**	**1.85E-03**	
Extracellular matrix glycoprotein	3	0.25	2.14E-03	

^• ^Number of genes, in the dataset of 57 genes, that map to the indicated Panther classification categories.

^•• ^Expected number of genes in the dataset of 57, based on the NCBI reference list of human genome.

^••• ^p-value as determined by the binomial statistic. A cutoff of 0.05 has been applied.

### Validation of microarray data by real-time RT-PCR

**Figure 3:**
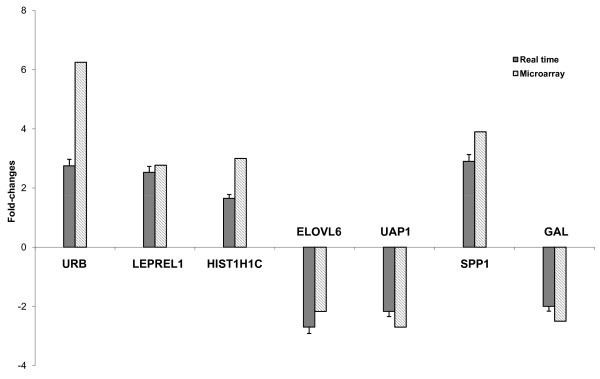
Real-time PCR validation of the microarray data, performed for 7 genes modulated by HCV. Total RNA from the 21-5 and 21-5 cured cell lines was used to assess mRNA levels using real time RT-PCR. Levels were normalized to cellular GAPDH; mRNA levels from 21-5 cured cells were set as the basis for the comparative results. Shown are the means (± SD) of three independent experiments. Fold-changes calculated for the microarray data are also indicated.

We performed real-time RT-PCR on 7 genes to validate the changes in gene expression observed by microarray analysis. They were selected from the 38 gene list because of high statistical significance and different expression levels [see Additional file 4]. The increase or decrease in expression of these genes by microarray analysis was in agreement with the real-time RT-PCR validation data (Fig. 3).

## Discussion

In the present work, we identified cellular genes which are modulated by HCV replication and protein expression by using a DNA microarray platform. This study represents the first global analysis that showed the sensitivity to reveal a common transcriptional response of Huh-7 cells to different clones of full-length HCV replicon. Specifically, we selected from the original datasets of differentially expressed genes, 38 genes that showed a concordant expression in three HCV replicon clones respect to both Huh-7 and IFN-α-cured cell lines.

Most of the 38 genes have never been described previously as relevant in HCV biology. To our knowledge, although modulation of SPP1, G1P2 and DDIT3 genes have been demonstrated in previous studies [34-37,25], the impact of HCV replication on the other 36 genes has never been reported. In addition, gene ontology analysis of the 38 genes revealed regulation by HCV of new biological processes like chromatin packaging and chromosome segregation as well as the identification of new genes involved in previously identified pathways such as lipid metabolism, extracellular matrix constitution and cytoskeletal network. The discovery of these new genes and gene families has been made possible through our microarray study and may constitute the basis for additional experiments useful to provide new insights into the biology of the virus.

Consistent with our microarray data, proteome analysis of cells harboring HCV replicon revealed a virus-induced perturbation in host cell protein synthesis, including proteins associated with host cytoskeleton, intracellular protein traffic, oxidative and ER stress and lipid metabolism [38,39]. Importantly, the down-regulation of enzymes involved in the mitochondrial ß-oxidation of fatty acids, such as carnitine palmitoyltransferase II (CPT2) and acyl-Coenzyme A dehydrogenase (ACADM), reported in proteome analysis [39] has been confirmed by our study (data not shown). This impaired mitochondrial function was also reported in a study describing a reduction in mitochondrial processes as a result of HCV-associated oxidative stress [40].

In a previous microarray analysis, the authors found that only 25 and 43 transcripts, respectively, differed in abundance by ≥ 2-fold (p ≤ 0.05) when two clones of HCV replicon were compared to the relative cured cell lines [26]. In contrast, we found that 288 genes were differentially expressed with a fold change ≥ 2 (p ≤ 0.05) when HCV replicon clone 21-5 was compared to the 21-5 cured cells. This discrepancy could be explained by the different sensitivity of the used microarray platforms. Indeed, the AB platform was reported to be more sensitive respect to the Affymetrix platform, detecting four times as many genes in an identical experimental design, and over seven times when additional technical replicates were included [27]. The higher sensitivity of the AB platform and the inclusion of four technical replicates for each samples, likely contributed to the increased number of differentially expressed genes that we detected.

Among the 38 selected genes modulated by HCV, we found that genes encoding DNA and RNA-binding proteins were significantly over-represented. Consistent with this observation, a larger proportion of genes encoding nucleic acid binding proteins have been found by microarray analysis in HCV-infected cirrhotic livers [41]. Interestingly, four genes encoding histone proteins were up-regulated in this gene set. Three of these genes (HIST2H2AC, HIST2H2AA and HIST1H1C) showed a fold induction ≥ 2 and a highly significant associated p-value in all comparisons [see Additional file 4]. Importantly, in microarray data analysis fold-change alone is widely considered an invalid statistic test and it needs to be associated with its p-value [42]. Therefore, the statistical significance of these results possibly suggests that modulation of histone genes represents a new HCV-induced pathway. To our knowledge, this is the first report coupling histone gene expression with HCV RNA replication. Interestingly, production of histone proteins is required to package DNA immediately upon initiation of DNA synthesis. As a consequence, transcription of histone genes takes place only at the onset of S-phase and it also respond to changes in rates of DNA synthesis [43,44]. Studies aimed at investigating HCV replication revealed that synthesis of HCV RNA is stimulated in S-phase cells [6,26]. Moreover, replication of viral RNA is regulated by the level of precursors of DNA synthesis, such as pyrimidine nucleosides, and/or the de novo synthesis of pyrimidines [45,46]. On this basis, we speculated that stimulation of nucleic acid metabolism, and consequently histone synthesis, by HCV may be an advantageous mechanism for replication of viral RNA. The induction of histone genes in HCV replicon cells further support the existence of a link between regulation of cell cycle and HCV RNA synthesis and requires further investigation.

As previously reported in HCV patients [41], genes encoding components of the extracellular matrix (ECM) were over-represented in the HCV gene set. The confirmation of *in vivo *data by microarray, clearly supports the biological significance of our study. One of these genes (SPP1) showed elevated levels of induction (fold > 3.9) associated with significant p-values [see Additional file 4]. Importantly, SPP1 and other genes implicated in the ECM turnover were up-regulated in the transition from mild to moderate fibrosis in HCV chronic patients and they have been proposed as new potential targets for antifibrosis drug development [34]. In addition, elevated serum levels of osteopontin, the protein encoded by SPP1, were found in HCV-associated B-cell nonHodgkin's lymphoma and type II mixed cryoglobulinemia [37]. Osteopontin is a secreted glycoprotein with a wide spectrum of biological activities promoting cell adhesion, migration, ECM invasion [47]. Evidences point to a central role for osteopontin in liver fibrosis as it induces hepatic stellate cell (HSC) migration and proliferation and stimulates synthesis of matrix metalloproteinase 2 (MMP-2) and type I collagen by HSC [48]. Consequently, modulation of SPP1 by HCV may be critical for liver fibrosis development and represents a potential target for therapy of HCV infection and/or disease progression.

In a recent report, it was shown that HCV RNA synthesis required an intact cytoskeleton, as inhibition of microtubule and actin polymerization resulted in decreased HCV replication [49]. Our microarray data indicated that three cytoskeletal genes (TUBB2A, CENPE, MTSS1) are significantly modulated by HCV replication. Tubulin beta 2A (TUBB2A), a structural constituent of microtubules, is up-regulated in HCV replicon cells as well as CENPE and MTSS1 genes both encoding for cytoskeletal proteins which bind to microtubule (CENPE) and actin (MTSS1) structures. The identification of these additional genes confirms the potential link between HCV RNA synthesis and cytoskeletal network.

Several evidences indicate that HCV induces alterations in lipid metabolism and contributes to the development of oxidative stress [13,14,40,50,33,11,12]. Consistent with this observation, in the dataset of 38 genes we found two genes, DDIT3 and ELOVL6, which are involved in oxidative stress induction and lipid biosynthesis, respectively.

Activation of the pro-apoptotic gene DDIT3, also known as Gadd153, by HCV has been previously demonstrated [35,36,15]. Elevated levels of Gadd153 protein in HCV replicon cells increased the sensitivity of these cells to oxidative stress suggesting that dysregulation of Gadd153 expression may be an important factor in HCV liver pathogenesis [36].

Recent reports suggest that increased synthesis of fatty acids enhances HCV replication [51]. More than 90% of fatty acids presents in cells possess chain lengths of 16 (palmitic acid) and 18 (stearic acid) carbons. The palmitic acid is synthesized by fatty acid synthase (FAS) in the cytosol. A high proportion of this palmitic acid (C16) is then converted to stearic acid (C18) in the endoplasmic reticulum (ER) by the enzyme, ELOVL6, which initiates the elongation of C16 to C18 fatty acids [52]. Interestingly, we found for the first time modulation of this gene in the HCV replicon cells. The robust down-regulation of ELOVL6 in our microarray dataset (fold change ≤ -2, p ≤ 0.008 in all comparisons) suggests that inhibition of this step would contribute to the accumulation of the upstream intermediate (palmitic acid), thus favouring HCV RNA synthesis. Although, it is now clear that lipid metabolism affects HCV replication, the role of ELOVL6 has not been investigated before and further experiments are needed to determine whether over-expression of ELOVL6 can inhibit HCV replication.

## Conclusion

Our microarray analysis provided an overview of alterations in gene expression induced by HCV even in different replicon clones. The resulting databases from microarray experiments contained additional information which may be useful for future investigations. Indeed, gene ontology classified HCV-modulated genes into functional categories which are relevant for viral biology and pathogenesis, such as immunity, oncogenesis, NF-kappaB cascade, lipid metabolism, extracellular matrix and cytoskeletal network. But, most importantly, these functional groups also contained genes which have not previously been reported to be impacted by HCV replication. In addition, we identified new functional categories such as histones, signalling molecules, select calcium binding proteins and cell motility as well as genes involved in Glutamine glutamate conversion and Blood coagulation pathways. Finally, we also provided a list of 38 genes which have been confirmed to be differentially expressed in all HCV clones. The future design of siRNAs directed against some of these genes might be useful to evaluate their importance in regulating HCV replication, providing an experimental background for selection of new antiviral drugs

## Methods

### Cell lines

The Huh-7 cells carrying the Sfl HCV full-length replicon (genotype 1b) were obtained from Dr. R. Bartenschlager. The cell lines that stably replicates the HCV replicon were the 21-5, 21-7 and 22-6 clones passaged as described [53,6]. Cured 21-5 replicon cells (21-5c) were obtained by treatment with 100U IFN-α/ml for 14 days, to eliminate self-replicating full-length HCV replicon. Clearance of replicon RNA was confirmed by RT-PCR and by loss of resistance to G418 [7]. HCV replicon cells were cultured in complete DMEM supplemented with 10% FCS, antibiotics, 1× non-essential amino acids, and 250 μg/ml (21-5, 21-7) and 500 μg/ml (22-6) G418 [53,6].

### AB expression array system analysis

Total RNA was extracted from 1× 10^6 ^cells using RNeasy kits (Quiagen) as described by the manufacturer. The quality of RNA was evaluated using the Agilent Bioanalyzer 2100 (Agilent Technologies). Only high quality RNA samples with a minimum RNA Integrity Number (RIN) value of 8 were considered for RNA labeling. One μg of total RNA from each sample was used to synthesize digoxigenin-UTP-labeled cRNA as described by the Applied Biosystems (AB) Chemiluminescent RT-IVT Labeling protocol. Array hybridization, array processing, chemiluminescence detection, image acquisition, and analysis were performed using AB Chemiluminescent Detection Kit and AB1700 Chemiluminescent Microarray Analyzer following manufacturer's protocol. These protocols are detailed in the Chemiluminescent Microarray analyzer Chemistry Guide (P/N 4338853) [54], Chemiluminescent Detection Kit (P/N 4339627) [55], and Chemiluminescent Microarray Analyzer User Guide (P/N 4338852B). The probe sequences are available at Panther website [56]. For each gene, the expression values were normalized across arrays by quantile normalization. "Detectable" calls for gene expression were based on Applied Biosystems recommendations (signal/noise ratio > 3.0 and FLAG values < 5000). A probe had to be called "detectable" in 75% of replicates in at least one tested sample group to be retained for further analysis. For each gene, expression fold-changes between groups were derived from the mean expression level in all replicates. P-values are derived from simple t-tests using log_2 _intensity data assuming equal variance. Hierarchical clustering analysis was performed in the Spotfire DecisionSite 9.1.1 (TIBCO Spotfire) software package, using the Unweighted Pair-Group Method with Arithmetic mean (UPGMA) as clustering method and Pearson's correlation as similarity measure.

Microarray data have been submitted to the ArrayExpress database [57]. Accession number for the data is E-MEXP-1686.

### Gene network pathway analysis

Gene Ontology (GO) annotations were analyzed with the Panther Protein Classification System [29] to identify functional annotations that were significantly enriched in this gene set compared to the entire human genome. Gene lists modulated by HCV were mapped onto biological pathways that were significantly represented.

### TaqMan assay validation

Total RNA was extracted from 1 × 10^6 ^cells using RNeasy kit (Qiagen) as described by the manufacturer and quantified by Bioanalyzer 2100 (Agilent Technologies). One hundred nanograms of total RNA was reverse transcribed using the high-capacity cDNA Archive Kit (Applied Biosystems), with random hexamer primers in a ABI Prism 7000 Sequence Detection System (Applied Biosystems) using the following thermal profile: 25°C for 10 min, 42°C for 1 h and 95°C for 5 min. PCR reactions were performed in triplicate on the ABI Prism 7000 Sequence Detection System (Applied Biosystems) using TaqMan chemistry with primer and probe sets from the Assay-on-Demand list (Applied Biosystems). The standard curve of each gene was compared to the standard curve of the housekeeping GAPDH gene and calculation of the slope of log [ng RNA] vs. ΔCt was always < 0.1. Fold induction was then calculated by ΔΔCt method [58] using GAPDH mRNA level to normalize values and the mRNA level of 21-5 cured cell line as a calibrator. Data are expressed as fold-changes.

## Authors' contributions

ARC had been involved in conception and design of the study, data analysis, interpretation and drafting the manuscript; CM carried out real-time PCR assays, most of microarray experiments and contributed to data interpretation; ET carried out cell culture experiments; PT, GG and AC participated in microarray experiments and data acquisition; AF performed statistical analysis of data; RB had been involved in data interpretation and revised the paper critically for important intellectual content; BD contributed to the revision of the paper critically; MR contributed to revise the paper. All the authors had given final approval of the version to be published.

## Supplementary Material

Additional file 1 Fold-changes (FC) and functional categories of 103 selected genes (104 probes) modulated by HCV. A table showing a list of 103 genes found to be modulated by HCV. For each gene, the table reports gene name, gene symbol, primary gene ID, fold-change in dataset 1 and in dataset 2, as well as the known molecular function or biological process. Click here for file

Additional file 2 Panther classification of biological processes, molecular functions and pathways significantly enriched in the set of 288 probes. A table showing biological processes, molecular functions and pathways found to be enriched in the set of 288 probes with fold-changes ≥ 2 from dataset 1 (21-5 cells *vs*. 21-5c cells). The number of observed and expected probes is reported, as well as the p-value. Click here for file

Additional file 3 Hierarchical clustering analysis of biological and technical replicates of Huh-7 cells and HCV clones (21-7, 22-6 and 21-5). The figure shows the intensity matrix plot ("heatmap") produced by hierarchical analysis of 725 probes modulated in HCV clones compared to Huh-7 cells (dataset 3, p ≤ 0.05). Each line represents one of the 725 probes. Each column represents a single array and is labeled by the name of the analysed sample followed (in bracket) by either number 1 or 2 distinguishing the two biological replicates and, in addition, a letter (a or b) distinguishing the two technical replicates, whenever performed. Click here for file

Additional file 4 Fold changes (FC) and functional categories of 57 selected genes (58 probes) modulated by HCV in all replicon clones. A table showing a list of 57 genes (58 probes) modulated by HCV in all analyzed replicon clones. Probes reported in bold were confirmed in all datasets. For each gene, the table reports gene name, gene symbol, primary gene ID, fold-change in dataset 1, dataset 2 and dataset 3, as well as the known molecular function or biological process. Click here for file
