# Supplementary material for: Microarray analysis identifies a common set of cellular genes modulated by different HCV replicon clones
Source: BMC Genomics. 2008 Jun 30;9:309. doi: 10.1186/1471-2164-9-309 (PMC2474623; doi:10.1186/1471-2164-9-309)
Supplement: Additional file 1 — Fold-changes (FC) and functional categories of 103 selected genes (104 probes) modulated by HCV. A table showing a list of 103 genes found to be modulated by HCV. For each gene, the table reports gene name, gene symbol, primary gene ID, fold-change in dataset 1 and in dataset 2, as well as the known molecular function or biological process. [file 1471-2164-9-309-S1.pdf]

Additional file 1. Fold-changes (FC) and functional categories of 103 selected genes (104 probes) modulated by HCV

Gene_Name	Gene_Symbol	Primary_Gene_ID *	ProbeID	FC (dataset 1)	FC (dataset 2)	Molecular Function|Biological Process
ornithine aminotransferase (gyrate atrophy)	OAT	4942	196523	-1.30	-1.60	Amino acid metabolism
solute carrier family 1 (glial high affinity glutamate transporter), member 3	SLC1A3	6507	148167	-2.00	-2.50	Amino acid metabolism|Cell communication
clusterin	CLU	1191	128174	1.45	1.22	Apoptosis
phosphoglycerate mutase 1 (brain)	PGAM1	5223	187172	-1.30	-1.25	Carbohydrate metabolism|Glycolysis
phosphorylase, glycogen; liver (Hers disease, glycogen storage disease type V	PYGL	5836	150387	-2.00	-1.60	Carbohydrate metabolism|Glycogen metabolism|
transaldolase 1	HSUP1|TALDO1	441951	229010	1.25	1.39	Carbohydrate metabolism|Pentose-phosphate shunt
latent transforming growth factor beta binding protein 1	LTBP1	4052	119769	1.97	2.89	Cell adhesion molecule|Extracellular matrix glycoprotein
cyclin D1	CCND1	595	141689	-1.60	-1.60	Cell cycle
catenin (cadherin-associated protein), alpha-like 1	CTNNAL1	8727	129321	-1.40	-1.40	Cell motility
thyroid hormone receptor interactor 6	TRIP6	7205	166985	1.73	1.53	Cell motility
paternally expressed 10	PEG10	23089	112068	-1.40	-1.40	Cell proliferation and differentiation
coiled-coil domain containing 80	URB	151887	111796	6.25	6.82	Developmental processes
secreted phosphoprotein 1 (osteopontin)	SPP1	6696	186825	3.93	20.31	Extracellular matrix
alpha-2-HS-glycoprotein	AHSG	197	186803	-2.50	-2.00	Extracellular matrix glycoprotein
leprecan-like 1	LEPREL1	55214	121792-134755 ▲	2.93	2.27	Extracellular matrix glycoprotein
coagulation factor C homolog, cochlin (Limulus polyphemus)	COCH	1690	189694	-1.40	-1.40	Extracellular matrix|Cell adhesion molecule
thrombospondin 1	THBS1	7057	162911	3.15	7.67	Extracellular matrix|Other extracellular matrix
insulin-like growth factor binding protein 3	IGFBP3	3486	104923	2.34	7.13	Homeostasis|Extracellular matrix protein-mediated signaling;
chloride intracellular channel 2	CLIC2	1193	147578	2.16	2.38	Homeostasis|Other homeostasis activities
ferritin, light polypeptide	FTL	2512	133972	1.50	1.46	Homeostasis|Other homeostasis activities
coagulation factor II (thrombin)	F2	2147	166635	-1.60	-1.60	Immunity and defense
monocyte to macrophage differentiation-associated	MMD	23531	211509	-1.60	-1.40	Immunity and defense|Lipid, fatty acid and steroid metabolism
complement component 5	C5	727	124482	-2.50	-1.40	Immunity and defense|Complement-mediated immunity
cathepsin E	CTSE	1510	172909	4.44	4.02	Immunity and defense|T-cell mediated immunity
haptoglobin	HP	3240	163480	-5.00	-5.00	Immunity and defenseexchange|Protein metabolism and modification
chromatin modifying protein 4A	CHMP4A	29082	190678	1.58	1.93	Intracellular protein traffic
mitochondrial GTPase 1 homolog (S. cerevisiae)	MTG1	92170	121062	-1.60	-1.60	Intracellular protein traffic
RALBP1 associated Eps domain containing 1	REPS1	85021	147893	1.45	1.36	Intracellular protein traffic|Endocytosis
mal, T-cell differentiation protein 2	MAL2	114569	194621	-1.60	-1.60	Intracellular protein traffic|General vesicle transport
ELOVL family member 6, elongation of long chain fatty acids	ELOVL6	79071	216339	-2.00	-2.50	Lipid fatty acid and steroid metabolism
apolipoprotein C-III	APOC3|LOC440838	345	212012	-2.50	-1.60	Lipid, fatty acid and steroid metabolism
diacylglycerol O-acyltransferase homolog 2 (mouse)	DGAT2	84649	204688	-1.40	-1.40	Lipid, fatty acid and steroid metabolism|Acyl-CoA metabolism
insulin induced gene 1	INSIG1	3638	215650	-2.50	-2.50	Lipid, fatty acid and steroid metabolism|Cholesterol metabolism
ATPase, Class V, type 10D	ATP10D	57205	137724	-2.50	-2.00	Lipid, fatty acid and steroid metabolism|Ion transport
Parkinson disease (autosomal recessive, early onset) 7	PARK7	11315	136848	1.65	1.35	Nucleic acid binding
staufen, RNA binding protein, homolog 2 (Drosophila)	STAU2	27067	151128	-2.00	-2.50	Nucleic acid binding
histone 1, H1c	HIST1H1C	3006	206232	3.11	2.23	Nucleic acid binding|Histone
histone 2, H2aa	HIST2H2AA	8337	139881-150267 ▲	3.40	2.33	Nucleic acid binding|Histone
histone 2, H2aa|histone 2, H2ac	HIST2H2AA|HIST2H2AC	8338	149647	3.33	2.23	Nucleic acid binding|Histone
histone 3, H2a	HIST3H2A	92815	209319	1.37	1.36	Nucleic acid binding|Histone
heterogeneous nuclear ribonucleoprotein D-like	HNRPDL	9987	158620	-1.60	-1.25	Nucleic acid binding|Ribonucleoprotein
interferon, alpha-inducible protein (clone IFI-15K)	G1P2	9636	133610	1.46	3.69	Nucleic acid binding|Ribosomal protein
mitochondrial ribosomal protein L13	MRPL13	28998	108144	-1.25	-1.40	Nucleic acid binding|Ribosomal protein
nucleolar protein family A, member 3	NOLA3	55505	189298	1.62	1.57	Nucleic acid binding|Ribosomal protein
N.A.	LOC401896	401896	201942	1.47	1.54	Nucleic acid binding|Ribosomal protein
SRY (sex determining region Y)-box 9	SOX9	6662	201106	1.34	1.28	Nucleic acid binding|Transcription factor
zinc finger protein 202	ZNF202	7753	176165	2.06	2.73	Nucleic acid binding|Transcription factor
eukaryotic translation initiation factor 2, subunit 2 beta, 38kDa	EIF2S2	8894	234929	1.26	1.25	Nucleic acid binding|Translation initiation factor|Translation factor
carbamoyl-phosphate synthetase 2	CAD	790	113678	1.32	1.61	Nucleoside, nucleotide and nucleic acid metabolism|Pyrimidine metabolism
pseudouridylate synthase-like 1	PUSL1	126789	142230	1.70	1.75	Nucleoside, nucleotide and nucleic acid metabolism|tRNA metabolism
melanoma antigen family A, 1 (directs expression of antigen MZ2-E)	MAGEA1	4100	117668	6.82	6.52	Oncogenesis|Other oncogenesis
melanoma antigen family A, 6|melanoma antigen family A, 3	MAGEA6|MAGEA3	4105	103778	57.95	58.46	Oncogenesis|Other oncogenesis
DNA-damage-inducible transcript 3	DDIT3	1649	163509	1.96	2.57	Oxidative stress
aldehyde dehydrogenase 3 family, member A2	ALDH3A2	224	171037	1.72	1.62	Oxidoreductase|Dehydrogenase
glutamate dehydrogenase 1	GLUD1	2746	178416	-2.50	-1.40	Oxidoreductase|Dehydrogenase
thioredoxin	TXN	7295	160960	1.20	1.31	Oxidoreductase|Other oxidoreductase
peroxiredoxin 4	PRDX4	10549	200038	1.38	1.40	Oxidoreductase|Peroxidase
dehydrogenase/reductase (SDR family) member 3	DHRS3	9249	125937	1.55	1.20	Oxidoreductase|Reductase|Dehydrogenase|Oxidoreductase

UDP-N-acteylglucosamine pyrophosphorylase 1	UAP1	6675	120981	-2.50	-2.50	Protein metabolism and modification|Protein glycosylation
serpin peptidase inhibitor, clade C (antithrombin), member 1	SERPINC1	462	107675	-2.50	-5.00	Protein metabolism and modification|Proteolysis
UDP-N-acetyl-alpha-D-galactosamine:polypeptide N-acetylgalactosaminyltrans	GALNT1	2589	150052	2.29	2.65	Protein metabolism and modification|Protein glycosylation
asparagine-linked glycosylation 12 homolog	ALG12	79087	112798	2.12	3.77	Protein metabolism and modification|Protein glycosylation
ring finger protein 144	RNF144	9781	101012	1.46	4.35	Proteolysis
pyroglutamyl-peptidase I	PGPEP1	54858	209677	1.81	1.89	Proteolysis
Myosin regulatory light chain	MRCL3	10627	195495	1.33	1.38	Select calcium binding protein
annexin A6	ANXA6	309	185083	-5.00	-5.00	Select calcium binding protein|Annexin
guanine nucleotide binding protein (G protein), beta polypeptide 1	GNB1	2782	206443	1.41	1.42	Signal transducrtion|Cell surface receptor mediated signal transduction
proprotein convertase subtilisin/kexin type 5	PCSK5	5125	178884	5.76	3.67	Signal transducrtion|Cell surface receptor mediated signal transduction
CD59 antigen p18-20	CD59	966	162611	1.83	1.83	Signaling molecule|Immunity and defense
galanin	GAL	51083	167511	-2.50	-3.30	Signaling molecule|Peptide hormone/Muscle contraction
chemokine (C-X-C motif) ligand 2	CXCL2	2920	173408	2.36	3.79	Signaling molecule|Chemokine
haptoglobin-related protein|chemokine (C-X-C motif) ligand 2	HPR|CXCL2|HP	3250	211417	-5.00	-5.00	Signaling molecule|Chemokine
ATP-binding cassette-ATG9 autophagy related 9 homolog A	ABCB6-ATG9A	10058-79065 ¶	209528	1.90	2.60	Transpor|Extracellular transport and import
eukaryotic translation initiation factor 4E nuclear import factor 1	EIF4ENIF1	56478	166561	-1.60	-1.25	unclassified
optineurin	OPTN	10133	129441	1.42	1.55	unclassified
transmembrane protein 63A	TMEM63A	9725	184048	-1.40	-1.60	unclassified
chromosome 15 open reading frame 41	C15orf41	84529	172419	2.05	1.77	unclassified
chromosome 18 open reading frame 10	C18orf10	25941	187311	1.70	1.75	unclassified
chromosome 18 open reading frame 21	C18orf21	83608	211326	1.95	1.88	unclassified
chromosome 1 open reading frame 148	C1orf148	574432	233980	2.16	3.44	unclassified
chondrosarcoma associated gene 1	CSAG1	158511	122812	2.66	2.84	unclassified
CSAG family, member 2	CSAG2	9598	106948	4.88	4.54	unclassified
growth arrest-specific 5	GAS5	60674	183660	1.43	1.91	unclassified
growth differentiation factor 15	GDF15	9518	182404	7.06	3.37	unclassified
nitric oxide synthase trafficker	NOSTRIN	115677	120791	1.98	3.78	unclassified
T-cell leukemia translocation altered gene	TCTA	6988	120662	1.66	1.33	unclassified
chromosome 21 open reading frame 6	C21orf6	10069	214481	-1.60	-2.00	unclassified
chromosome 2 open reading frame 33	C2orf33	56947	163168	1.56	1.21	unclassified
solute carrier family 30 (zinc transporter), member 9	SLC30A9	10463	123127	-2.00	-2.50	unclassified
signal transducer and activator of transcription 3 interacting protein 1	STATIP1	55250	169477	1.84	1.53	unclassified
zinc finger protein 364	ZNF364	27246	186169	1.17	1.91	unclassified
ring finger protein 190	RNF190	162333	197739	1.44	1.42	unclassified
N.A.	C3F	10162	204279	-1.60	-1.60	unclassified
N.A.	RP3-462O23.2	90529	209110	1.94	3.19	unclassified
N.A.	KIAA1706	80820	201203	1.39	2.97	unclassified
N.A.	LA16c-360B4.1	64788	161532	2.21	2.52	unclassified
N.A.	N.A.	hCG1811496.1	154676	3.35	6.53	unclassified
N.A.	N.A.	hCG2007929	228667	2.50	2.41	unclassified
N.A.	N.A.	hCG1643350.2	102706	1.67	2.16	unclassified
N.A.	N.A.	hCG1738432.2	104959	2.28	2.00	unclassified
N.A.	N.A.	hCG2006634.1	234451	1.48	1.76	unclassified
N.A.	N.A.	hCG2041466	134996	-1.60	-1.60	unclassified

N.A.: not assigned

* IDs starting with hCG are from human Celera Genomics database

▲ Two different probes identify the same gene.FC corresponding to the first probe are shown.

¶ Two different genes are identified by the same probe
