# Supplementary material for: Microarray analysis identifies a common set of cellular genes modulated by different HCV replicon clones
Source: BMC Genomics. 2008 Jun 30;9:309. doi: 10.1186/1471-2164-9-309 (PMC2474623; doi:10.1186/1471-2164-9-309)
Supplement: Additional file 2 — Panther classification of biological processes, molecular functions and pathways significantly enriched in the set of 288 probes. A table showing biological processes, molecular functions and pathways found to be enriched in the set of 288 probes with fold-changes ≥ 2 from dataset 1 (21-5 cells vs. 21-5c cells). The number of observed and expected probes is reported, as well as the p-value. [file 1471-2164-9-309-S2.pdf]

Additional file 2. Panther classification of Biological processes, Molecular functions and Pathways significantly enriched in the set of 288 probes

	Observed Probes•	Expected Probes••	p-value•••	p-value with Bonferroni correction
**Biological Process**				
**Lipid, fatty acid and steroid metabolism**	**23**	**8.66**	**2.63E-05**	**8.17E-04**
Steroid hormone metabolism	8	0.51	6.25E-08	1.24E-05
Steroid metabolism	12	2.06	1.55E-06	2.25E-04
Fatty acid metabolism	7	2.1	5.70E-03	
**Developmental processes**	**36**	**24.2**	**1.11E-02**	
Skeletal development	5	1.38	1.34E-02	
Mesoderm development	11	6.2	4.93E-02	
**Amino acid metabolism**	**7**	**2.59**	**1.63E-02**	
Amino acid catabolism	6	0.56	2.60E-05	3.77E-03
**Carbohydrate metabolism**	**13**	**6.66**	**1.78E-02**	
Other polysaccharide metabolism	8	1.6	2.41E-04	3.50E-02
**Ion transport**	**14**	**6.93**	**1.08E-02**	
Anion transport	6	1.01	6.10E-04	
**Blood circulation and gas exchange**	**4**	**1**	**1.88E-02**	
Other blood circulation and gas exchange activity	2	0.2	1.79E-02	
**Ligand-mediated signaling**	**10**	**4.73**	**2.22E-02**	
Other receptor mediated signaling pathway	6	2.36	3.28E-02	
**Intracellular signaling cascade**	**16**	**9.8**	**3.92E-02**	
MAPKKK cascade	6	2.04	1.75E-02	
JNK cascade	3	0.69	3.23E-02	
**Immunity and defense**	**22**	**14.82**	**4.35E-02**	
Granulocyte-mediated immunity	3	0.72	3.64E-02	
**Other metabolism**	**15**	**6.29**	**1.91E-03**	
**Protein metabolism and modification**				
Proteolysis	19	10.8	1.32E-02	
**Sulfur metabolism**	**4**	**1.05**	**2.17E-02**	
**Molecular Function**				
**Extracellular matrix**	**14**	**4.32**	**1.43E-04**	**4.14E-03**
Extracellular matrix glycoprotein	7	1.25	3.02E-04	4.86E-02
Other extracellular matrix	3	0.42	8.75E-03	
**Oxidoreductase**	**17**	**6.78**	**5.71E-04**	**1.65E-02**
Oxygenase	4	1.14	2.82E-02	
Oxidase	3	0.76	4.23E-02	
**Signaling molecule**	**20**	**8.94**	**7.88E-04**	**2.28E-02**
Growth factor	6	1.41	3.17E-03	
**Transferase**	**19**	**9.94**	**5.86E-03**	
Glycosyltransferase	9	2.76	2.06E-03	
**Select regulatory molecule**				
Protease inhibitor	6	**1.45**	**3.69E-03**	
Serine protease inhibitor	4	0.85	1.12E-02	
Kinase inhibitor	4	0.48	1.53E-03	
**Nucleic acid binding**				
Histone	5	0.97	3.11E-03	
**Cell junction protein**				
Gap junction	2	0.21	1.98E-02	
**Ligase**				
Other ligase	5	1.64	2.57E-02	
**Transfer/carrier protein**				
Apolipoprotein	2	0.26	2.81E-02	
**Lyase**				
Cyclase	2	0.27	3.04E-02	
**Cell adhesion molecule**				
Other cell adhesion molecule	4	1.3	4.32E-02	
**Ion channe**				
Anion channel	2	0.34	4.55E-02	
**Select calcium binding protein**				
Annexin	3	0.8	4.70E-02	
**Pathways**				
Glutamine glutamate conversion	3	0.06	2.78E-05	3.65E-03
Oxidative stress response	4	0.77	7.98E-03	
**Blood coagulation**	**3**	**0.62**	**2.46E-02**	
Plasminogen activating cascade	2	0.24	2.36E-02	

•Number of probes, in the dataset of 288, that map to the indicated Panther classification categories

••Expected number of probes, in the dataset of 288, based on the NCBI reference list of human genome

•••p-value as determined by the binomial statistic. A cutoff of 0.05 has been applied
