# Supplementary figures and images for: Microarray analysis identifies a common set of cellular genes modulated by different HCV replicon clones
Source: BMC Genomics. 2008 Jun 30;9:309. doi: 10.1186/1471-2164-9-309 (PMC2474623; doi:10.1186/1471-2164-9-309)

## Slide 1
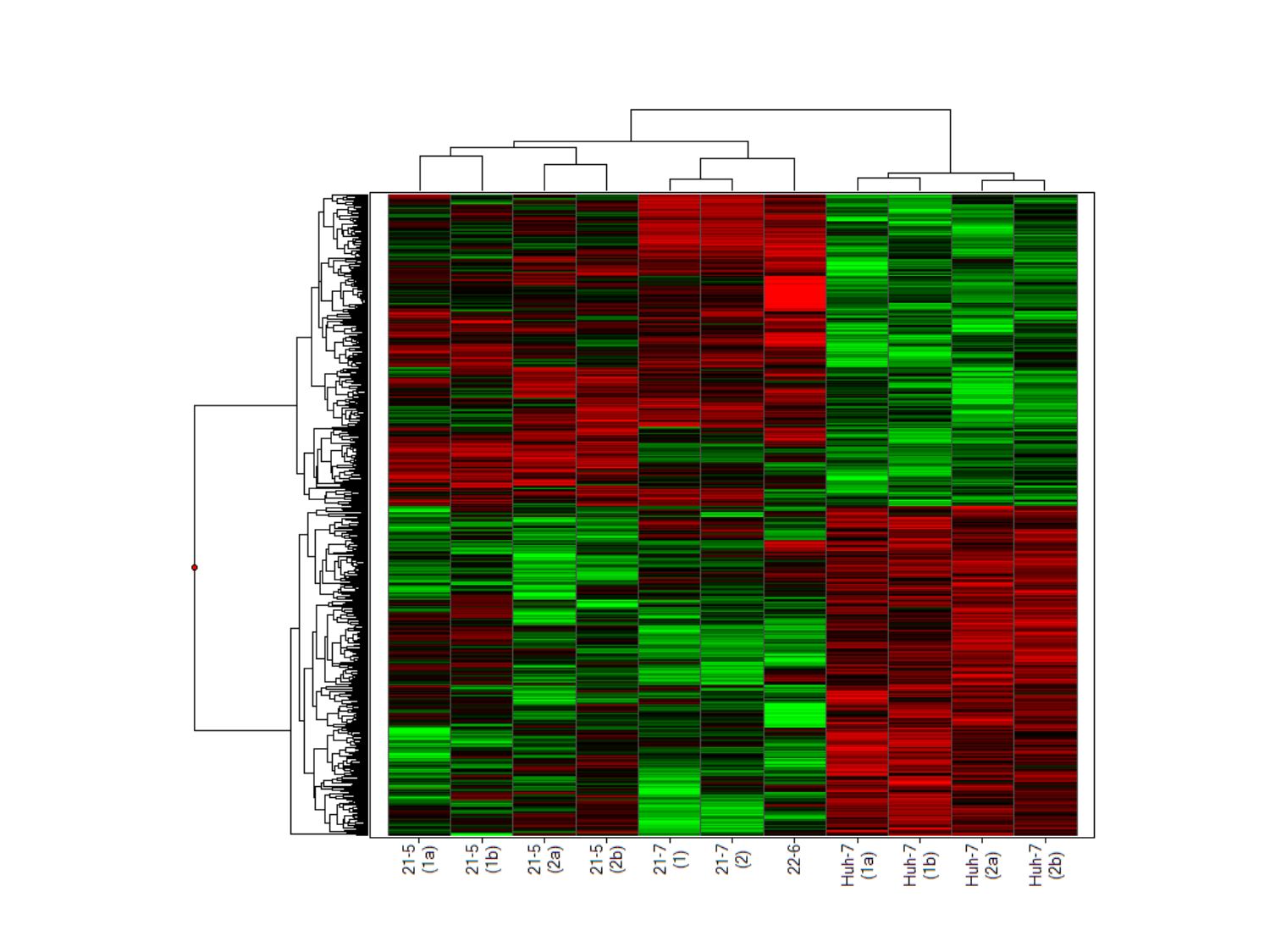

Supplement: Additional file 3 — Hierarchical clustering analysis of biological and technical replicates of Huh-7 cells and HCV clones (21-7, 22-6 and 21-5). The figure shows the intensity matrix plot ("heatmap") produced by hierarchical analysis of 725 probes modulated in HCV clones compared to Huh-7 cells (dataset 3, p ≤ 0.05). Each line represents one of the 725 probes. Each column represents a single array and is labeled by the name of the analysed sample followed (in bracket) by either number 1 or 2 distinguishing the two biological replicates and, in addition, a letter (a or b) distinguishing the two technical replicates, whenever performed. [file 1471-2164-9-309-S3.ppt]
