# Supplementary material for: Microarray analysis identifies a common set of cellular genes modulated by different HCV replicon clones
Source: BMC Genomics. 2008 Jun 30;9:309. doi: 10.1186/1471-2164-9-309 (PMC2474623; doi:10.1186/1471-2164-9-309)
Supplement: Additional file 4 — Fold changes (FC) and functional categories of 57 selected genes (58 probes) modulated by HCV in all replicon clones. A table showing a list of 57 genes (58 probes) modulated by HCV in all analyzed replicon clones. Probes reported in bold were confirmed in all datasets. For each gene, the table reports gene name, gene symbol, primary gene ID, fold-change in dataset 1, dataset 2 and dataset 3, as well as the known molecular function or biological process. [file 1471-2164-9-309-S4.pdf]

Additional file 4. Fold-changes (FC) and functional categories of 57 selected genes (58 probes) modulated by HCV in all replicon clones

Gene_Name	Gene_Symbol [#]	Primary_Gene_ID *	ProbeID	FC (dataset 1)	p-value	FC (dataset 2)	p-value	FC (dataset 3)	p-value	Molecular Function|Biological Process
secreted phosphoprotein 1 (osteopontin)	**SPP1**	6696	186825	3.93	0.04424	20.31	0.00081	9.02	0.00471	Extracellular matrix
leprecan-like 1	**LEPREL1**	55214	121792-134755 [▲]	2.93	0.00190	2.27	0.01008	2.51	0.00773	Extracellular matrix glycoprotein
alpha-2-HS-glycoprotein	**AHSG**	197	186803	-2.77	0.01289	-1.80	0.03191	-1.64	0.01521	Extracellular matrix glycoprotein
coagulation factor C homolog, cochlin (Limulus polyphemus)	**COCH**	1690	189694	-1.44	0.04389	-1.37	0.01590	-1.56	0.00068	Extracellular matrix|Cell adhesion molecule
histone 2, H2aa|histone 2, H2ac	**HIST2H2AA|HIST2H2AC**	8338	149647	3.33	0.00084	2.23	0.00154	3.01	0.00098	Nucleic acid binding|Histone
histone 2, H2aa	**HIST2H2AA**	8337	139881-150267 [▲]	3.40	0.00451	2.33	0.01219	2.71	0.00105	Nucleic acid binding|Histone
histone 1, H1c	**HIST1H1C**	3006	206232	3.11	0.02024	2.23	0.00682	2.36	0.01426	Nucleic acid binding|Histone
histone 1, H2ab	HIST1H2AB	8335	162382	2.14	0.02288	1.42	0.11108	1.78	0.03605	Nucleic acid binding|Histone
histone 3, H2a	**HIST3H2A**	92815	209319	1.37	0.02435	1.36	0.04659	1.59	0.00715	Nucleic acid binding|Histone
interferon, alpha-inducible protein (clone IFI-15K)	**G1P2**	9636	133610	1.46	0.03502	3.69	0.00014	2.40	0.00317	Nucleic acid binding|Ribosomal protein
nucleolar protein family A, member 3	**NOLA3**	55505	189298	1.62	0.00016	1.57	0.01657	1.53	0.02152	Nucleic acid binding|Ribosomal protein
mitochondrial ribosomal protein L18	MRPL18	29074	197989	1.31	0.04743	1.22	0.15189	1.39	0.02632	Nucleic acid binding|Ribosomal protein
mitochondrial ribosomal protein L13	**MRPL13**	28998	108144	-1.25	0.04667	-1.40	0.02570	-1.40	0.01744	Nucleic acid binding|Ribosomal protein
ribosomal protein L15	RPL15	6138	211819	-1.40	0.03461	-1.10	0.47122	-1.60	0.04747	Nucleic acid binding|Ribosomal protein
Parkinson disease (autosomal recessive, early onset) 7	**PARK7**	11315	136848	1.65	0.00256	1.35	0.02759	1.53	0.00272	Nucleic acid binding
SRY (sex determining region Y)-box 9	**SOX9**	6662	201106	1.34	0.01193	1.28	0.03671	1.48	0.00487	Nucleic acid binding|Transcription factor
heterogeneous nuclear ribonucleoprotein D-like	**HNRPDL**	9987	158620	-1.60	0.00369	-1.25	0.03770	-1.25	0.00145	Nucleic acid binding|Ribonucleoprotein
eukaryotic translation initiation factor 3, subunit 6 interacting proteir	EIF3S6IP	51386	147314	-1.25	0.04855	-1.25	0.05087	-1.25	0.03227	Nucleic acid binding|Translation initiation factor
staufen, RNA binding protein, homolog 2 (Drosophila)	**STAU2**	27067	151128	-2.00	0.02005	-2.50	0.00713	-2.00	0.00243	Nucleic acid binding
carbamoyl-phosphate synthetase 2	**CAD**	790	113678	1.32	0.03305	1.61	0.03762	1.76	0.01649	Nucleoside, nucleotide and nucleic acid metabolism|Pyrimidine metabolism
pseudouridylate synthase-like 1	**PUSL1**	126789	142230	1.70	0.03460	1.75	0.01421	1.74	0.00984	Nucleoside, nucleotide and nucleic acid metabolism|tRNA metabolism
N.A.	LOC392347	392347	189484	-2.00	0.00645	-1.25	0.07531	-1.42	0.00752	Nucleoside, nucleotide and nucleic acid metabolism|Purine metabolism
chromatin modifying protein 4A	**CHMP4A**	29082	190678	1.58	0.00412	1.93	0.01251	1.90	0.01692	Intracellular protein traffic
lectin, mannose-binding 2-like	LMAN2L	81562	184532	1.34	0.02258	1.14	0.23656	1.43	0.03010	Intracellular protein traffic|Protein targeting
RALBP1 associated Eps domain containing 1	**REPS1**	85021	147893	1.45	0.01041	1.36	0.01581	1.35	0.00207	Intracellular protein traffic|Endocytosis
tubulin, beta 2A	TUBB2A	7280	194068	2.05	0.02892	1.17	0.16997	1.24	0.01343	Intracellular protein traffic|Chromosome segregation
mal, T-cell differentiation protein 2	**MAL2**	114569	194621	-1.60	0.00297	-1.60	0.00059	-1.40	0.00664	Intracellular protein traffic|General vesicle transport
mitochondrial GTPase 1 homolog (S. cerevisiae)	**MTG1**	92170	121062	-1.60	0.00042	-1.60	0.00036	-1.60	0.00498	Intracellular protein traffic
ornithine aminotransferase (gyrate atrophy)	**OAT**	4942	196523	-1.30	0.04518	-1.60	0.00401	-2.00	0.00049	Amino acid metabolism
phosphoglycerate mutase 1 (brain)	**PGAM1**	5223	187172	-1.30	0.00741	-1.25	0.02002	-1.30	0.01724	Carbohydrate metabolism|Glycolysis
centromere protein E, 312kDa	CENPE	1062	165425	1.91	0.01026	1.42	0.05624	1.44	0.01774	Cytoskeletal protein|Microtubule binding motor protein
metastasis suppressor 1	MTSS1	9788	188654	-2.50	0.02671	-2.00	0.06249	-2.50	0.00163	Cytoskeletal protein|Actin binding cytoskeletal protein
coiled-coil domain containing 80	**URB**	151887	111796	6.25	0.00400	6.82	0.00428	4.37	0.00869	Developmental processes
OAF homolog (Drosophila)	OAF	220323	121408	-2.50	0.01014	-1.25	0.21529	-2.00	0.02764	Developmental processes|Neurogenesis
ELOVL family member 6, elongation of long chain fatty acids	**ELOVL6**	79071	216339	-2.00	0.00836	-2.50	0.00104	-2.00	0.00426	Lipid fatty acid and steroid metabolism
DNA-damage-inducible transcript 3	**DDIT3**	1649	163509	1.96	0.02985	2.57	0.00182	2.07	0.00474	Oxidative stress
WW domain containing E3 ubiquitin protein ligase 1	WWP1	11059	191676	-1.60	0.02962	-1.40	0.07106	-1.42	0.00621	Protein metabolism and modification|Proteolysis
UDP-N-acteylglucosamine pyrophosphorylase 1	**UAP1**	6675	120981	-2.50	0.04612	-2.50	0.04547	-2.50	0.01566	Protein metabolism and modification|Protein glycosylation
serpin peptidase inhibitor, clade C (antithrombin), member 1	**SERPINC1**	462	107675	-2.50	0.04040	-5.00	0.00556	-3.30	0.00258	Protein metabolism and modification|Proteolysis
proprotein convertase subtilisin/kexin type 5	**PCSK5**	5125	178884	5.76	0.00085	3.67	0.00367	3.27	0.00664	Signal transducrtion|Cell surface receptor mediated signal transduction
guanine nucleotide binding protein (G protein), beta polypeptide 1	**GNB1**	2782	206443	1.41	0.03384	1.42	0.03691	1.69	0.00407	Signal transducrtion|Cell surface receptor mediated signal transduction
guanine nucleotide binding protein (G protein), gamma 5	GNG5	2787	178569	1.41	0.03747	1.32	0.11380	1.50	0.04230	Signal transducrtion|Cell surface receptor mediated signal transduction
calmodulin binding transcription activator 1	CAMTA1	23261	164871	1.57	0.02239	1.29	0.15521	1.43	0.04496	Signal transduction|Calcium mediated signaling
CD59 antigen p18-20	**CD59**	966	162611	1.83	0.01405	1.83	0.00271	1.48	0.04245	Signaling molecule|Immunity and defense
galanin	**GAL**	51083	167511	-2.50	0.01756	-3.30	0.00823	-3.30	0.00004	Signaling molecule|Peptide hormone/Muscle contraction
ATP-binding cassette-ATG9 autophagy related 9 homolog A	**ABCB6-ATG9A**	10058-79065 [¶]	209528	1.86	0.01649	2.56	0.01220	2.74	0.00516	Transport|Extracellular transport and import
phosphatidylinositol transfer protein, membrane-associated 1	PITPNM1	9600	128232	1.77	0.00536	1.19	0.12377	1.61	0.01931	unclassified
ring finger protein 190	**RNF190**	162333	197739	1.44	0.03907	1.42	0.04236	1.50	0.00244	unclassified
optineurin	**OPTN**	10133	129441	1.42	0.03900	1.55	0.02416	1.41	0.04741	unclassified
transmembrane protein 63A	**TMEM63A**	9725	184048	-1.40	0.03327	-1.60	0.00117	-1.40	0.00187	unclassified
COMM domain containing 8	COMMD8	54951	127990	-1.60	0.04959	-1.60	0.05728	-1.40	0.02605	unclassified
transmembrane protein 70	TMEM70	54968	173256	-1.25	0.03320	-1.10	0.33313	-1.60	0.04281	unclassified
cell division cycle associated 7	CDCA7	83879	207416	-1.60	0.03533	-1.40	0.10852	-1.60	0.01646	unclassified
eukaryotic translation initiation factor 4E nuclear import factor 1	**EIF4ENIF1**	56478	166561	-1.60	0.00258	-1.25	0.01037	-1.60	0.00966	unclassified
erythrocyte membrane protein band 4.1-like 1	EPB41L1	2036	122347	-2.00	0.00480	-2.00	0.07714	-2.00	0.04108	unclassified
leucine rich repeat and coiled-coil domain containing 1	LRRCC1	85444	200352	-3.30	0.01610	-1.42	0.34595	-2.50	0.04805	unclassified

[#] Genes showing a FC with p ≤ 0.05 in all three datasets (1, 2 and 3) are reported in bold; FC of the other genes showed p ≤ 0.05 in dataset 1 and 3
N.A.: not assigned
[▲] Two different probes identify the same gene. FC corresponding to the first probe are shown.
[¶] Two different genes are identified by the same probe
